# Functionalization of Osteoplastic Material with Human Placental Growth Factor and Assessment of Biocompatibility of the Resulting Material In Vitro

**DOI:** 10.3390/pharmaceutics16010085

**Published:** 2024-01-08

**Authors:** Diana Ya Aleynik, Andrey E. Bokov, Irina N. Charykova, Yulia P. Rubtsova, Daria D. Linkova, Ekaterina A. Farafontova, Marfa N. Egorikhina

**Affiliations:** Federal State Budgetary Educational Institution of Higher Education, Privolzhsky Research Medical University of the Ministry of Health of the Russian Federation, 10/1 Minin and Pozharsky Square, 603005 Nizhny Novgorod, Russia; daleynik@yandex.ru (D.Y.A.); andrei_bokov@mail.ru (A.E.B.); irina-ch0709@yandex.ru (I.N.C.); rubincherry@yandex.ru (Y.P.R.); linckovadaria@yandex.ru (D.D.L.); ekaterina_farafontova@mail.ru (E.A.F.)

**Keywords:** osteoplastic material, placental growth factor, adipose-derived stromal stem cells, biocompatibility

## Abstract

This article provides the results of a study of the interaction of placental growth factor with adipose-derived stem cells (ASCs) of various origins, as well as the possibility of generating osteoplastic material based on xenogeneic matrix functionalization with human placental growth factor (PLGF). It is demonstrated that the greatest release of this factor from the functionalized material into the medium occurs during the first 3 h of contact with the model medium, but then the levels of the factor being released fall sharply, although release did continue throughout the 7 days of observation. The modified material was not cytotoxic, and its surface provided good cell adhesion. During 3 days of cultivation, the ASCs proliferated and migrated more actively on the surfaces of the modified material than on the surfaces of the control material. This study can serve as the basis for the development of original methods to functionalize such osteoplastic material by increasing PLGF immobilization by creating stronger bonds in order to regulate both factor dosage and the dynamics of the factor release into the environment. Further studies in experimental animals should facilitate assessment of the effectiveness of the functionalized materials. Such studies will be useful in the development of osteoplastic materials with new properties resulting from the inclusion of growth factors and in research on their biological activity.

## 1. Introduction

Currently, the problem of restoring bone defects that form in connection with diseases and injuries of various etiologies remains an urgent matter. An important reason for the actualization of this problem is the increase in the proportion of the elderly population and the consequent increases in the incidence of degenerative pathology and osteoporosis. Specific attention is given here to one of the most socially significant localizations that leads to severe health impairments—degenerative lesions of the spine. For example, according to the results of the published meta-analysis, the incidence of clinically significant degenerative spinal stenosis is 11.0% with a 95.0% confidence interval of 4.0–18.0% [1,2]. It is known that this pathology is one of the most common indications for surgical treatment, and in the case of spine segment instability, stabilization of the segment with bone grafting and various fixation techniques is required in addition to decompression [3,4,5,6]. Another clear trend is the increase in the incidence of both high-energy trauma and pathological fractures of the spine because of osteoporosis [7,8]. In the case of an unstable spinal injury or a pathological fracture, surgical interventions using transpedicular fixation and interbody fusion are also required [9,10,11]. The above-mentioned trends are resulting in an increasing frequency of decompression and stabilization interventions on the spine.

In the case of stabilizing surgical interventions on the spine, as well as other complex orthopedic procedures, the bone tissue’s regenerative potential and its mechanical properties are of the utmost importance. The success of surgical interventions largely depends on regulation of the recovery processes, which, according to many professionals, is associated with the complex nature of the processes of bone tissue formation mediated by coordinated mechanisms of direct and indirect osteogenesis. Indirect osteogenesis is mainly associated with angiogenesis [12,13], whereas implementation of processes of both direct and indirect osteogenesis is attributable to complex systems of growth factors [14].

Currently, the important role of such growth factors systems in the process of bone tissue restoration is not in doubt, but the complexity of relations between specific factors, and the significance of each of them within the long-term and multi-stage process of bone regeneration are still the subject of ongoing research. Based on newly obtained experimental data, studies are being conducted into the possibility of including certain growth factors in the complex treatment of bone defects.

The use of bone replacement materials with modified properties to accelerate bone block formation is a strategy that can help reduce the incidence of complications during the treatment of bone tissue defects. It is generally accepted that an ideal osteoplastic material should have such properties as osteogenicity, osteoinductivity, osteoconductivity, and the capacity to osteointegrate without forming a delimiting capsule around the graft [15,16]. An increase in osteoinductivity can be achieved by saturating the bone replacement material with growth factors that induce the migration of stem cells, their differentiation into osteoblasts, and graft vascularization [17]. Currently, the effects of such factors as BMP-2 and VEGF are the most studied from among the numerous growth factors involved in the processes of osteogenesis [18].

Bone morphogenetic protein 2 (BMP-2) is considered the most potent direct osteogenic growth factor. Recombinant BMP-2 is approved by the US Food and Drug Administration and has already been used clinically [19,20,21,22]. However, clinical experience has shown that a really significant dose of BMP-2 is required for an effective therapeutic outcome since this protein stimulates osteoregeneration only at high concentrations [23,24], while its half-life is only 7 min in vivo [24,25].

The use of high doses of BMP-2 can, unfortunately, result in both high costs and side effects. It has also become apparent that the use of BMP-2 can trigger an excessive inflammatory response, edema, and heterotopic ossification [18,26,27,28]. Moreover, there is information that the use of this factor can contribute to the growth of malignant neoplasms [29,30]. There have also been negative reports of immune reactions, of renal failure, and of supraventricular arrhythmia as a result of the administration of BMP-2 [31,32,33]. It should be emphasized that the use of BMP-2 also requires a consideration of the patient’s age because age directly affects the biological potential of many growth factors. Furthermore, the impact of BMP-2 on bone regeneration cannot always be controlled. For example, there have been reports of observations of ineffective osteogenesis following BMP-2 administration. Some publications also demonstrate that this cytokine can facilitate stem cell differentiation into adipocytes, thereby increasing the content of fat cells in the resulting bone tissue, ultimately resulting in a decrease in the concentration of bone trabeculae and to bone tissue lysis [34,35,36]. These numerous side effects have stimulated the search for alternatives to BMP-2 and have drawn attention to the factors mediating indirect osteogenesis.

Such factors are the cytokines that are regulators of angiogenesis and that are an integral part of the vascular endothelial growth factor (VEGF) system. These factors include five cytokines: VEGF-A, VEGF-B, VEGF-C, VEGF-D, and placental growth factor (PLGF). VEGF-A is the major and most studied of the vascular endothelial factors [37]. Many studies have also been devoted to strategies aimed at researching the possibility of using the cytokines of this group in order to improve bone tissue regeneration [38]. It is known that necrosis and hypoxia are the primary issues in the pathogenesis of bone tissue damage, while VEGF is required for the formation of a normal vascular network in areas of tissue damage [39,40]. It has been shown that, where grafts containing VEGF have been introduced into the area of a bone defect, vascularization is enhanced, bone mass can increase by 1.6–2 times, and osseointegration of the xenografts is improved [39,41,42]. However, there have also been papers reporting that, in the case of the introduction of grafts containing VEGF into defect areas, the only effect registered was an increase in angiogenesis, without any increase in the bone mass [17]. A possible reason for such contradictory results may be due to the kinetics of the specific factor’s release from the carrier and, perhaps, differences in the VEGF doses used. It seems likely that it is necessary to ensure a gradual and prolonged release of VEGF, to prevent hyperstimulation from causing angiomas or the generation of capillaries without connections to the vascular bed [43].

A possible method to overcome these shortcomings is to choose alternative growth factors that enhance osteoregeneration at lower doses.

The attention of researchers has been drawn to proangiogenic factors and, specifically, to placental growth factor—PLGF, which, as mentioned above, belongs to the family of vascular endothelial growth factors (VEGFs) and has a particular role in angiogenesis [44,45,46,47,48]. While the importance of VEGFs in both the direct and indirect acceleration of osteogenesis was established and studied long ago, the importance of PLGF is less well known [46,47]. It has been demonstrated that PLGF stimulates the formation of collaterals in ischemia and enhances the impact of VEGF [48]. Numerous PLGF functions are described in the literature, such as the ability to stimulate and activate numerous cell types, including bone marrow-derived cells [44,48,49,50,51].

A number of studies have demonstrated the role of this cytokine in the regulation of osteogenesis and angiogenesis in response to mechanical stress during bone tissue regeneration [52,53,54]. It has been shown that the expression of the PLGF gene in stem cells depends on the intensity and duration of exposure to mechanical stress [23]. A number of studies reached the conclusion that PLGF has a positive chemotactic effect in endochondral osteogenesis, whereas blocking the receptor for this cytokine slows bone formation [55,56,57,58]. It has been established that PLGF has a significant role at all stages of bone tissue regeneration. During the initial stage, PLGF stimulates the osteogenic differentiation of stem cells, and later it promotes bone tissue remodeling by stimulating osteoclast differentiation and angiogenesis [59].

A potential advantage of this cytokine is that it promotes pro-osteogenic differentiation of mesenchymal stem cells; here the maximum pro-osteogenic effect is observed at a low concentration of 10–25 ng/mL [23]. The effect of PLGF is dose-dependent; for instance, at a concentration of over 50 ng/mL, osteoclast differentiation and activation are promoted, together with activation of angiogenesis in the form of capillary development from endothelial cells [23,59]. An additional advantage of the use of PLGF is that its osteogenic effect is seen at a significantly lower concentration than that needed with recombinant BMP-2 [58].

The above-mentioned properties encourage consideration of researching the promising use of PLGF and its interactions with osteoplastic material for the formation of functionalized materials with new properties. The use of materials that include growth factors should improve the effectiveness of bone tissue regeneration, in particular in patients at risk of non-union after bone grafting.

The most important condition that ensures successful formation and functioning of a material loaded with growth factors is the choice of base, i.e., the matrix. Such material must be tested through practice, accessible, and easy to use. Materials based on xenobone are of special interest among the types of material widely employed in clinical practice, especially those used in maxillofacial surgery. Processed xenografts have become a powerful alternative to transplant materials of human origin, primarily due to their lower production costs and the greater availability of raw materials [60]. Processed xenomaterials based on bovine tissues are considered to be the most similar to regenerated human bone grafts, being second only to autografts, as well as being safe products that can be used in clinical practice, when bone regeneration is required in reconstructive operations [61,62]. For instance, European dentists still prefer xenografts as bone substitutes due to their clinical predictability [63]. Considering the above, a marketed and clinical-use approved xenogeneic, osteoplastic, non-demineralized matrix based on bovine cancellous bone was chosen as the PLGF-carrier for our study.

The purpose of this study was to consider the possibility of modifying xenogeneic osteoplastic material incorporating human PLGF, the impact of PLGF on cultures of adipose-derived stem cells (ASCs) of various origins, and the in vitro biocompatibility of such functionalized material.

## 2. Materials and Methods

### 2.1. Human Placental Growth Factor

This study was conducted using rhPLGF recombinant protein—a human placental growth factor (from the CHO cell line that produces rhPLGF, SciStoreLab LLC, Moscow, Russia). The protein was used in the form of a sterile lyophilized white powder, packaged in 2 μg doses. For our research, a PLGF solution was prepared at a concentration of 200 ng/mL in phosphate buffer (pH—7.2–7.4; 0.2 M), and then the available concentration was adjusted to 20 ng/mL.

### 2.2. Osteoplastic Matrix

**Figure 1:**
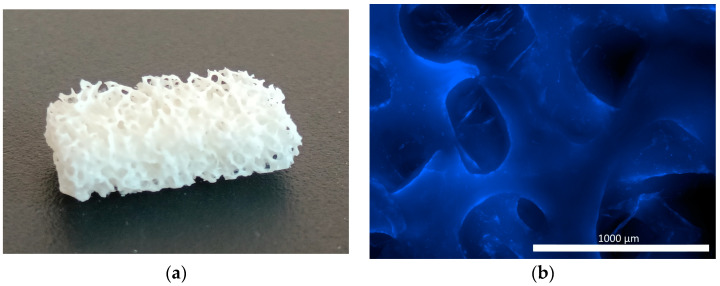
Osteoplastic non-demineralized matrix before its introduction into the experiment: (**a**) external view of the matrix; (**b**) porous matrix structure without cells (stained with fluorochrome BD Pharmingen™ Hoechst 33342 Solution, BD Biosciences™, Franklin Lakes, NJ, USA).

A commercially available osteoplastic non-demineralized matrix, which is approved for clinical use, was used as the matrix. This is bovine bone tissue treated physically and chemically, and then sterilized using the gas method. As a natural osteoplastic material, formed on the basis of spongy and cortical bone tissue with collagen; this material has been designed to fill bone defects. The research was conducted using block-shaped samples of the material, the size of which was selected depending on the objectives of the study. The material samples were white, solid and durable, and had clearly visible pores (Figure 1a,b).

### 2.3. ASC Cultures

Human and rabbit ASCs were used as test cultures.

### 2.4. Obtaining Human ASC Cultures

Human ASCs were obtained from samples of waste adipose tissue collected during planned liposuction surgeries. Each donor of biological material provided his/her informed voluntary consent for the use of their material in research. The donors of biological material were examined for vector-borne infections; they had no history of cancer, tuberculosis, or autoimmune diseases.

The sampling of biological material and obtaining cell cultures for their subsequent use in in vitro studies were conducted in line with the guidelines of the Declaration of Helsinki and approved by the local ethics committee of the FSBEI HE PRMU MOH Russia (Nizhny Novgorod, Russia, 30 June 2023, MoM No. 09).

The adipose tissue samples were collected under sterile, operating room conditions into transport medium (medium 199 or Hanks’ solution with penicillin/streptomycin antibiotics, PavEko LLC, Moscow, Russia) and transferred to the laboratory. The cells were isolated by thermal enzymatic treatment for one hour at 37 °C, using Type I collagenase solution (Stemcell Technologies, Vancouver, BC, Canada). The cultivation was conducted in Costar plastic (USA) using a special serum-free MesenCult MSK basal medium and supplement (Stemcell Technologies, Canada) with antibiotics (penicillin/streptomycin) as a growth medium. Cultivation was performed in a CO_2_ incubator at 37 °C, 5% CO_2_, and absolute humidity.

### 2.5. Obtaining Rabbit ASC Cultures

All procedures for working with animals were conducted in a vivarium in compliance with the requirements of the European Convention for the Protection of Vertebrate Animals used for Experimental and Other Scientific Purposes (Strasbourg, France, 2006). The work with animals was approved by the local ethics committee of the FSBEI HE PRMU MOH Russia (Nizhny Novgorod, Russia; 21 January 2022, MoM No. 1).

Rabbit ASCs were obtained from adipose tissue biopsies, which were collected from the groin or withers of examined, healthy animals under operating room vivarium conditions after anesthetized euthanasia (1.0 “XilaVet”, Pharmamagist Ltd., Budapest, Hungary and 1.0 “Zoletil”, Virbac Sante Animale, Carros, France). The adipose tissue biopsies were placed in vials with sterile Hanks’ solution with a 5-fold set of antibiotics (penicillin-streptomycin) and transferred to the laboratory. Further work with the rabbit material was similar to the manipulations used to obtain human ASCs. To cultivate rabbit ASCs, α-MEM growth medium was used with the addition of 10% fetal bovine serum (FBS), glutamine, and antibiotics (penicillin/streptomycin). Cultivation was conducted at absolute humidity, 37 °C, 5% CO_2_, and the medium was replaced twice a week. Media and reagents from PanEco LLC (Moscow, Russia) and Costar plastic vessels (Washington, DC, USA) were used. The culture was subcultured when it reached a 70% subconfluent monolayer.

Cell concentrations were calculated using a CountTess automatic cell counter (Invitrogen by Thermo Fisher Scientiﬁc, Waltham, MA, USA). Trypan blue intravital dye was used to determine the percentage of viable cells.

All cultures of human and rabbit ASCs for this study were obtained and characterized in the biotechnology laboratory of the University Clinic of the FSBEI HE PRMU MOH Russia. All were sterile and not contaminated with mycoplasmas or viruses. Before their introduction into the experiment and at all stages of the study, the culture cells were morphologically homogeneous, being fibroblast-like cells with clear contours, pronounced processes, and dense nuclei. The cells formed a subconfluent monolayer (70–80%) with a distinctive curl pattern. Cell viability in the cultures was 97–98%. Cultures of 3–4 passages were used in the experiments.

### 2.6. Microscopy

The growth dynamics of the cell cultures on plastic were regularly monitored using light and phase-contrast microscopy (Leica DMI 3000 B inverted microscope, Leica Microsystems, Germany, LAS v. 4.3 software, Leica Microsystems, Wetzlar, Germany) and stored in a video archive for subsequent analysis. To visualize cells on the studied material samples, fluorescence microscopy was carried out on a Cytation 5 imager (BioTek, Winooski, VE, USA), and the microphotographs also stored in the video archive.

### 2.7. Assessment of the Impact of Placental Growth Factor on ASC Cultures

Human ASC cultures were removed from the plastic surface with a 0.25% trypsin solution in Versene and seeded into 18 flasks (25 cm^2^, Costar, USA) with a density of 10 × 10^3^/cm^2^. Typical fibroblast-like cells could be visualized on all flasks 24 h after repeated seeding; they were evenly distributed over the plastic surface. The flasks with cultures were divided into two series of 9 flasks each: an experimental series and a control series. When the cells were well spread over the plastic, 24 h after repeated seeding, the medium in all flasks was completely replaced. A medium with a 10% solution of placental growth factor in phosphate buffer (4.5 mL of MesenCult + supplement medium and 500 μL of a solution of placental growth factor) was added to the flasks of the experimental series, bringing the final concentration of PLGF to 20 ng/mL. The control series flasks were supplemented with the same medium with 10% phosphate buffer (4.5 mL of MesenCult + supplement growth medium + 500 μL of phosphate buffer with a pH of 7.2–7.4).

At test periods (24, 48, 72 h) after the start of exposure to the placental growth factor, the state of the culture on 3 flasks of the control series and 3 flasks of the experimental series was recorded and stored in the video archive. Then, medium samples were taken from each flask to identify the growth factors present, and aliquoted into microtubes, while the cells were removed from the plastic, and the density and viability of the cells obtained from each flask were calculated. The number of cells in the suspension was counted using a Goryaev’s chamber (5 fields of view) and/or a CounTess automatic cell counter (Invitrogen by Thermo Fisher Scientiﬁc, Waltham, MA, USA) in association with a trypan blue staining exclusion test solution.

Rabbit ASC cultures were used to study the impact of human placental growth factor on experimental animal cells. The experiments were conducted similarly to the experiments with the human ASC cultures, but cultivation was performed using α-MEM medium with the addition of 2% glutamine and 10% TES to the growth medium.

At test periods (24, 48, 72 h) after the start of exposure to the placental growth factor, the state of the culture on 3 flasks of the control series and 3 flasks of the experimental series was recorded and stored in the video archive. Then the cells were removed from the plastic with a 0.25% trypsin solution in Versene and the density and viability of the cells in each flask were calculated.

### 2.8. Enzyme-Linked Immunosorbent Assay (ELISA)

At the same test periods, samples of the conditioned medium were aliquoted in 500 μL volumes, transferred into sterile microtubes, and stored at −20 °C. The contents of fibronectin and VEGF-A were determined in samples of the conditioned medium using the ELISA method. The following kits were used for analysis: Thermo Fisher Scientiﬁc (Bender MedSystems, Vienna, Austria) for VEGF-A and Technoclone (Technoclone Herstellung von Diagnostika und Arzneimitteln GmbH, Vienna, Austria) for fibronectin. Optical density measurements were conducted on an INFINITI F50, Tecan photometer (Tecan Austria GmbH, Grödig, Austria), using Magellan™ software (https://lifesciences.tecan.com/software-magellan), which enabled automatic building of a calibration curve and determination of the substance concentration.

### 2.9. Functionalization of the Xenogeneic Human PLGF Matrix and Assessment of Protein Release into the Medium In Vitro

To functionalize the PLGF matrix, a solution of the factor in phosphate buffer (pH = 7.2–7.4) with a concentration of 20 ng/mL was prepared and sterilized by filtration. Nine sterile samples of osteoplastic matrix with dimensions of 0.8 × 0.8 × 0.4 cm were fully immersed in the prepared factor solution for 90 min. After 90 min, the samples were removed from the solution and dried in a laminar flow unit for 24 h. After drying, the matrix samples were placed into the wells of a 24-well plate, and then each sample was covered with 2 mL of phosphate buffer. The plate with samples was placed in a CO_2_ incubator at 37 °C, 5% CO_2_, and high humidity. After 3 h of release, the extract was taken from all wells, aliquoted into microtubes, and stored at −20 °C. Then, 2 mL of fresh phosphate buffer was added to all sample wells and the plate was placed in a CO_2_ incubator for 24 h. After 24 h, the medium from the wells was carefully removed and 2 mL of fresh phosphate buffer was added. After 3 h of exposure, the extract was sampled, aliquoted, and stored at −20 °C. Then the samples were covered with fresh phosphate buffer. Similar manipulations were repeated after 3 and 7 days to determine the amount of PLGF in the model medium, extracted over each 3 h period. Thus, the amount of factor extracted over each 3 h at various times after the start of the release process (the beginning of the interaction of the samples with the model medium) was determined.

The dynamics of the PLGF content differences in the model medium in the experimental and control series were assessed by the enzyme-linked immunosorbent assay method using R&D Systems kits (Bio-Techne^®^, Minneapolis, MN, USA). This study was conducted in line with the manufacturer’s recommendations. Measurements were performed using an INFINITI F50, Tecan photometer (Tecan Austria GmbH, Grödig, Austria), with Magelan software.

### 2.10. Assessment of Cytotoxicity of the PLGF-Functionalized Osteoplastic Matrix

The introduction of a growth factor into an osteoplastic material can change its properties and, specifically, the toxicity of the modified material. Therefore, to characterize a material with new properties, it is necessary to assess the initial level of its cytotoxicity; this was done using the standard MTT assay. The MTT assay is based on the capacity of living cells to reduce yellow 3-(4,5-dimethylthiazol-2-yl)-2,5-tetrazolium bromide (MTT) into purple intracellular MTT-formazan crystals, soluble in isopropanol or dimethyl sulfoxide (DMSO). The amount of the reduced product is measured photometrically at a wavelength of 540 nm. A decrease in the optical density of the experimental samples compared to the control samples, which is recorded on a tablet reader, allows rendering a conclusion about the cytotoxic impact of the test substance on the cells [64].

A culture of human dermal fibroblasts of passage 4–5 was used as a test culture; it was obtained in the biotechnology laboratory of the University Clinic of the FSBEI HE PRMU MOH, Russia. The culture was sterile and not contaminated with mycoplasmas or viruses. The viability of the cells in culture before entering the experiment was 97%.

The test sample containers were filled with DMEM medium with 1% antibiotics (penicillin/streptomycin) and 2% fetal calf serum on Day 1 and Day 7 to obtain an extract, and then placed in a CO_2_ incubator under standard conditions. After 1 day or 7 days, the extract above the samples was taken and dilutions (1:1; 1:2; 1:4; 1:8) were prepared. Cells at a concentration of 1 × 10^5^/mL were seeded onto the wells of a flat-bottomed 96-well plate in DMEM medium with 1% antibiotics and 10% inactivated fetal calf serum, and cultured at 37 °C, 5% CO_2_, and absolute humidity for 3 days. After 3 days of cultivation, the growth medium above the cells was replaced in the control series with fresh growth medium, and in the experimental series, with either whole extract or one of its dilutions. There were 8 wells for each series. After 24 h of cultivation, the medium in all wells was replaced with the MTT solution, and then the cells were incubated with it for 4 h. The MTT solution was prepared at a concentration of 1 mg/mL in Hanks’ balanced solution. After 4 h of incubation, the supernatant was carefully removed, DMSO (200 μL) was added, and then the optical density (OD) was recorded at 540 nm using an INFINITI F50 photometer, Tecan (Tecan Austria GmbH, Grödig, Austria).

The relative growth rate was determined as a percentage using the following formula:

$$RGR(\%)=\frac{mean\;OD\;of\;test\;compound}{mean\;OD\;of\;control}\times 100$$

where RGR is the relative growth rate, and OD is the optical density.

The intensity of the relative growth rate was assessed on the basis of a scale for cytotoxicity assessment [65]. Cytotoxic grades 0 and 1 were considered to indicate no cytotoxicity (RGR, 100–75%), Cytotoxic grade 2 (74–50%) as low cytotoxicity, Cytotoxic grade 3 (49–25%) as medium cytotoxicity, and Cytotoxic grades 4 and 5 (24–0%) as high cytotoxicity.

### 2.11. Assessment of Adhesion, Proliferation, and Migration of Human ASCs in Osteoplastic Matrix Samples

Nine samples of osteoplastic matrix loaded with PLGF (experimental series) and 9 samples of matrix with no added growth factor (control series) were put in the wells of a 24-well plate. Human ASCs were seeded onto the matrix samples, at 200 thousand cells per well in 2 mL of MesenCult growth medium (MSK basal medium and supplement, Stemcell Technologies, Canada) with antibiotics (penicillin/streptomycin) added. At the test periods of 24 h (Day 1); on Day 3 and Day 7, 3 matrix samples from the experimental series and 3 samples from the control series were taken; the migration of cells into the matrix and the number of cells that had grown on the samples were analyzed. During the study, quantitative assessment of changes in the number of cells was conducted according to the original method [66]. The method is based on intravital staining of nuclei using fluorochrome Hoechst 33342 (BD Biosciences™, Franklin Lakes, NJ, USA; excitation wavelength of 377 nm and emission wavelength of 447 nm), wide-field fluorescence microscopy in 10 fields of view using the Z-stack function, and subsequent counting of cell nuclei on the cross-linked Z-stack microphotographs. The total number of cells per 1 mm^3^ was estimated. For that, after each test period, the studied matrix samples were stained with fluorochromes, the cells on the matrix were photographed, the photos saved in the photo archive, and a quantitative analysis was performed. Marking and assessment of the proportion of dead cells in the structure of the material during the cultivation process were made using the TO-PRO™3 Ready Flow™ Reagent Invitrogen™ (Invitrogen by Thermo Fisher Scientific, Waltham, MA, USA) fluorochrome, which stains only the nuclei of dead cells (excitation wavelength of 586 nm, emission wavelength of 647 nm). This fluorochrome is characterized by its specificity for double-stranded DNA molecules, but also its inability to penetrate the cytoplasm of viable cells. Descriptions of the viable cells adhering to the material and their morphology were obtained using intravital staining of the cell cytoplasm with Calcein AM (BD Pharmingen™, Franklin Lakes, NJ, USA). This dye functions in association with esterase activity, which is only typical of viable cells (excitation wavelength of 495 nm, emission wavelength of 515 nm). The fluorochrome staining was conducted in line with the manufacturers’ protocols.

The assessment of cell migration within the structure of the material was made on the basis of the original method [67]. To implement the method, photographic recording of the distribution of the cells at various depths in the structure of the samples was conducted in 12 fields of view using layer-by-layer shooting along the *Z* axis to the visualization depth from the first (on the surface) to the deepest-located cell (within the material)—to register the positions of the nuclei within the depth of the structure.

Quantitative analysis and assessment of the depth of cell penetration into the sample structure were performed by means of wide-field fluorescence microscopy on a Cytation 5 imager (BioTek, Winooski, VE, USA) using Gen 5 Image+ software.

### 2.12. Statistical Analysis

Statistical analysis was performed using the STATISTICA 6.0 (Dell Technologies Inc., Round Rock, TX, USA) software system. This study was conducted using methods of nonparametric statistics, including the Mann–Whitney test and the Wilcoxon paired comparison test.

## 3. Results

### 3.1. Interaction of PLGF with ASCs

The primary requirement was to assess the impact of human PLGF on the properties of cultured ASCs over time. The interaction of PLGF with cultures of human ASCs and ASCs of an experimental animal—rabbit—was assessed.

#### 3.1.1. Interaction of PLGF with Human ASCs

**Figure 2:**
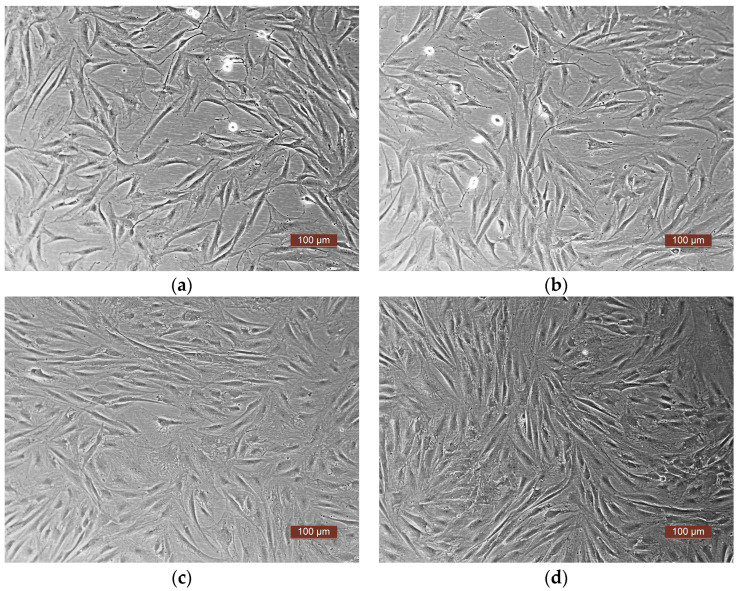
Representative microphotographs of the interaction of human ASC cultures with PLGF and control buffer (phase contrast). (**a**,**c**) control; (**b**,**d**) experiment (PLGF); (**a**,**b**) 24 h after the beginning of the study; (**c**,**d**) 72 h after the beginning of the study.

Visual microscopic assessment of the interaction of human PLGF with human ASC cultures revealed dynamic growth of the culture on the plastic culture vessel surfaces, with maintenance of the typical fibroblast-like cell morphology. Twenty-four hours after the beginning of interaction in both series (experimental and control), a large number of dividing cells was seen in all cultures, and by 72 h after the introduction of PLGF or control buffer, in all experiments, a thick confluent monolayer had been formed both in the control and experimental series (Figure 2).

Under visual observation, no differences were seen between the control and experimental series in all three experiments, either in the state of the cells or in the nature of the monolayer during cultivation. Thus, adding human PLGF to the growth medium did not damage the human ASCs or interfere with their adhesion, spreading, or proliferation on plastic.

Quantitative analysis over time showed the cell density changing differently in the various experiments. In Experiments No. 1 and No. 3, the ASCs proliferated actively for up to 48 h, with the proliferation being greater in the experimental series. However, during the period from 48 to 72 h of observation in Experiment No. 1, the cell density did not change, this most likely being due to the reverse inhibition phenomenon. In Experiment No. 3, by 72 h, the cell density in both series had increased significantly, but it was even for the series. In Experiment No. 2, cells had actively proliferated in both series during the entire observation period, although the proliferation was greater in the experimental series (Table 1).

The results demonstrated the absence of any negative impact of PLGF on the morphology, adhesion, or proliferation of human ASCs grown on plastic. Moreover, statistically significant differences in cell density were recorded for the experimental and control series in all three experiments by 48 h after the beginning of interaction with the factor, and in Experiment No. 2, the effect continued up to 72 h of observation.

To assess the impact of PLGF on the secretory activity of human ASCs, the following were identified: accumulation in the conditioned culture medium of the main proangiogenic factor VEGF-A (Table 2) and of fibronectin—one of the main components of the extracellular matrix (Table 3).

Assessment of the protein content in the conditioned media indicated that in Experiment No. 1, accumulation of both proteins was intensive and increased by 3–4 times during the study period. In Experiment No. 2, after 24 h of cultivation, the level of VEGF in the medium was slightly lower than in Experiment No. 3. During cultivation in Experiment No. 2, the level of VEGF increased, whereas in Experiment No. 3 in the experimental series, this increase was slightly greater than in the control series. However, the differences between the experimental and control series were not statistically significant. The content of fibronectin 24 h after the beginning of interaction with PLGF for the cultures in Experiments No. 2 and No. 3 was significantly higher than in Experiment No. 1. However, the content of fibronectin in Experiments No. 2 and No. 3 in the conditioned media during the observation period increased less than in Experiment No. 1, and was approximately similar in both the experimental and control series.

Thus, the data (Table 2 and Table 3) show that the introduction of human PLGF into the growth medium did not impact the secretory activity of human ASCs in culture.

#### 3.1.2. Interaction of Human PLGF with Rabbit ASCs

The impact of human PLGF on rabbit ASCs was studied because further use of osteoplastic materials requires preclinical in vivo studies based on experiments in animal models. Rabbits are widely used as experimental animals in regenerative medicine [68,69,70,71]. It is known that rabbit bones are similar to human bones in their biochemical properties [72], while their sizes are sufficient to study osteoplastic materials.

**Figure 3:**
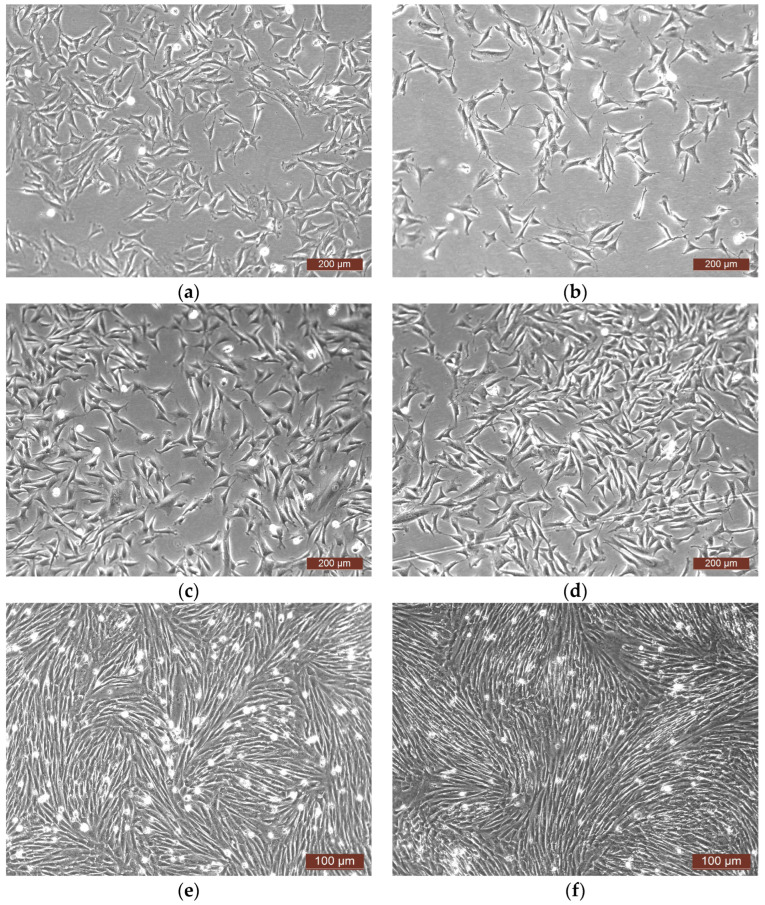
Representative photographs of rabbit ASCs (phase contrast). (**a**,**c**,**e**) control; (**b**,**d**,**f**) experiment (PLGF); (**a**,**b**) 24 h after the start of the study; (**c**,**d**) 48 h after the start of the study; (**e**,**f**) 72 h after the start of the study.

During our study of the impact of human PLGF on rabbit ASCs, intense proliferation of these cells was recorded both in the experimental series and in the control series. As a rule, 24 h after repeated seeding, the rabbit ASCs formed clones of various densities on the plastic, proliferated rapidly, and by one day after the introduction of PLGF, or of control medium, a large number of dividing cells and the beginning of fusion of the clones could be seen (Figure 3a,c). After 48 h, the rabbit ASCs had formed a subconfluent monolayer on the plastic surface and were continuing to divide actively (Figure 3c,d). After 72 h, a dense confluent monolayer could be visualized in all control and experimental series; however, there were practically no dividing cells, but a significant number of floating “rounded” cells could be seen in the medium (Figure 3e,f).

Quantitative analysis demonstrated that the dynamics of the changes in cell densities of the rabbit ASC and human ASC cultures were slightly different. In Experiments No. 1r and No. 2r, active proliferation of the cells on plastic (Table 4, Experiment No. 1r and No. 2r) was accompanied by greater changes in cell density throughout the entire observation period in the experimental series, with an approximately four-fold overall increase by 72 h of cultivation. In Experiment No. 3r, the cells proliferated actively only up to 48 h; their density had increased by approximately 3–4 times in both series. Further, over the next 24 h, the cell density remained at the same level in both the experimental and control series. During this period in Experiment No. 3r, the differences in cell density for the experimental series compared with that in the control series were insignificant (Table 4, Experiment No. 3r).

The studies demonstrated that the introduction of human PLGF into the growth medium did not have a negative impact on the state of the cells or the properties of the rabbit ASC monolayers. The cells proliferated more actively after interaction with PLGF in the experimental series for up to 48 h in all experiments, and in two of them, the introduction of PLGF into the growth medium resulted in active stimulation of proliferation of the ASCs in culture up to 72 h of observation. This was manifested in an increase in cell density in the experimental series compared to the control series when cultivated with the growth factor. The results of Experiment No. 3r demonstrated active proliferation and statistically significant differences between the experimental and control series during the first 48 h after the factor was introduced. During the subsequent cultivation in Experiment No. 3r, the cell density in both the experimental and control series remained practically unchanged, meaning that the differences in cell density between the experimental and control series persisted.

These recorded effects of human PLGF on rabbit ASCs indicate that studies of the effectiveness and safety of modified materials containing human PLGF are worthwhile in a rabbit bone defect model.

Considering the above results, one can say that there is no negative impact of PLGF on the morphological properties and viability of either human or rabbit ASCs while growing in culture. In experiments with the various ASC cultures, a significant stimulation of proliferation was noted during the first 48 h after the contact when interacting with human PLGF; further activation of the process was not evidenced by all the experiments. No negative impact was seen when studying the accumulation of proteins in the conditioned medium of human ASC cultures interacting with human PLGF.

### 3.2. Dynamics of PLGF Release from Functionalized Osteoplastic Matrix Samples

The next stage of the work was dedicated to the assessment of the dynamics of human PLGF release over a fixed period (3 h) after its incorporation into osteoplastic matrix samples for test points at intervals over 7 days in a model environment. During the 7 days of this study, human PLGF was extracted from the matrix into the model medium (Table 5). The experiment demonstrated that the maximum release of the factor occurred in the first 3 h of the study, whereas at the next test period—after 24 h—the amount of factor released into the model environment was almost an order of magnitude smaller. With the later test intervals, the factor release into the medium showed a slower decrease.

The results indicate that the impregnation of the matrix with PLGF allows the factor to survive within the matrix structure with some being released into the model medium over the 7 days of the study. One can assume that this method of applying PLGF to the matrix (impregnation) enables the factor to be absorbed into the surface of the matrix but without strong interactions forming between the matrix material and the factor; thus, a massive release (washing out) of the factor is seen during the first 3 h of interaction after the samples were transferred into the liquid medium.

### 3.3. Assessment of the Cytotoxicity of Matrix Samples Functionalized with PLGF

The most important characteristic of any medical material is its lack of toxicity. Any modification of the material could result in a change in its properties and therefore requires testing of the modified material for cytotoxicity. The cytotoxicity of the PLGF material was assessed using a standard MTT assay. Neither extracts, nor dilutions of these, of the medium around the material taken after two periods of release (at Day 1 and Day 7) demonstrated any pronounced cytotoxicity (Table 6). As the data in Table 6 show, both the extract and its dilutions were of Cytotoxic grade 1 (non-cytotoxic), and only one of the dilutions from Day 7 of release was Cytotoxic grade 2 (low cytotoxicity). Such properties are acceptable for bone replacement materials.

### 3.4. Adhesion, Proliferation, and Migration of Human ASCs in Functionalized Matrix Samples

**Figure 4:**
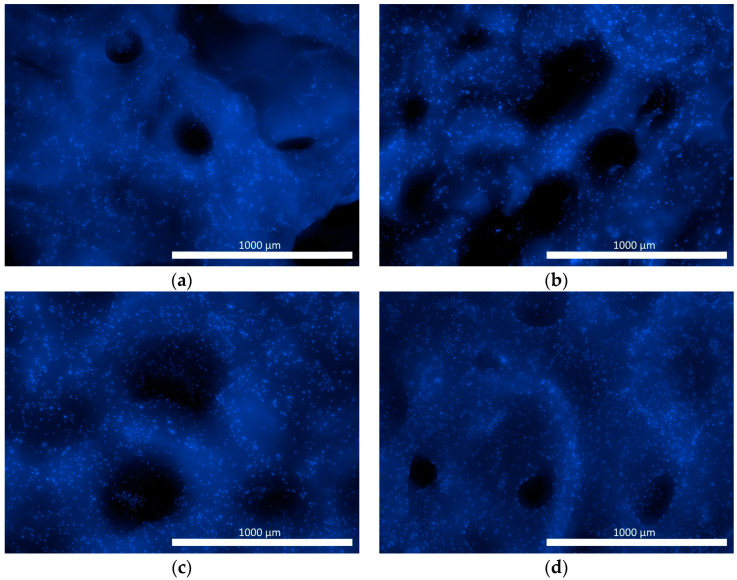
Representative photographs of the nuclei of ASCs within the matrix samples (fluorescence microscopy, Hoechst fluorochrome 3334). (**a**,**b**) 1 day after seeding cells onto the samples; (**c**,**d**) 7 days of cell culturing on the samples; (**a**,**c**) control series; (**b**,**d**) experimental series.

When ASCs were cultured in osteoplastic matrix samples, evident cell adhesion was recorded in both the experimental and control (no factor) samples (Figure 4).

**Figure 5:**
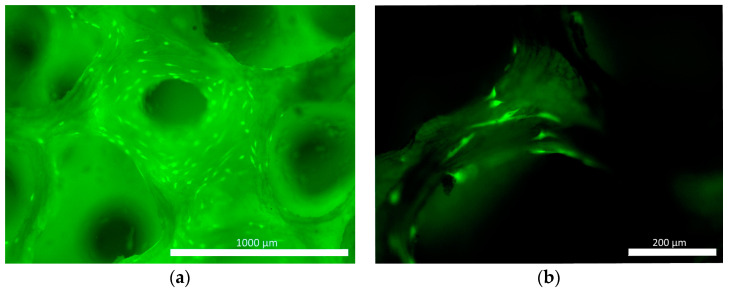
Representative photographs of ASCs on the PLGF-functionalized matrix (fluorescence microscopy, Calcein AM fluorochrome). (**a**) The structure of the material and the cells adhering to the structural elements; (**b**) ASCs with characteristic morphology on the matrix.

The use of intravital calcein dye allowed recording of both the preservation of the viability of the ASCs in the matrix containing the factor and the ability of the cells to spread across the surface of the structural elements of the material with a typical fibroblast-like morphology (Figure 5). The results confirmed the cytocompatibility of the material with the test culture and the ability of its surface topography to provide conditions for cell adhesion and survival.

**Figure 6:**
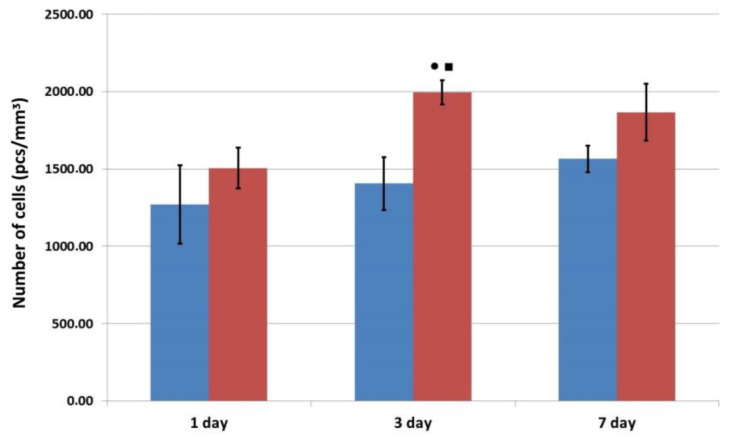
Changes in the number of cells during cultivation of human ASCs in samples of osteoplastic matrix. Note: 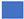
—control series, 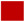
—experimental series; ●—*p* < 0.05 compared with Day 1; ■—*p* < 0.05 comparison, control v experiment (Wilcoxon rank sum test).

Quantitative analysis demonstrated that in the case of a PLGF-functionalized matrix, a more intense proliferation of ASCs was recorded compared to the proliferation of cells on a matrix with no factor (Figure 6). In both series, the number of cells in the matrix increased greatly in the first three days after repeated seeding, but then it remained practically unchanged. Here, the number of cells that died during cultivation in the matrix samples, did not exceed two percent, although in the matrix samples containing no factor, there had been a slight increase to Day 7, while in the matrix samples with the factor, there was no change in the level.

**Figure 7:**
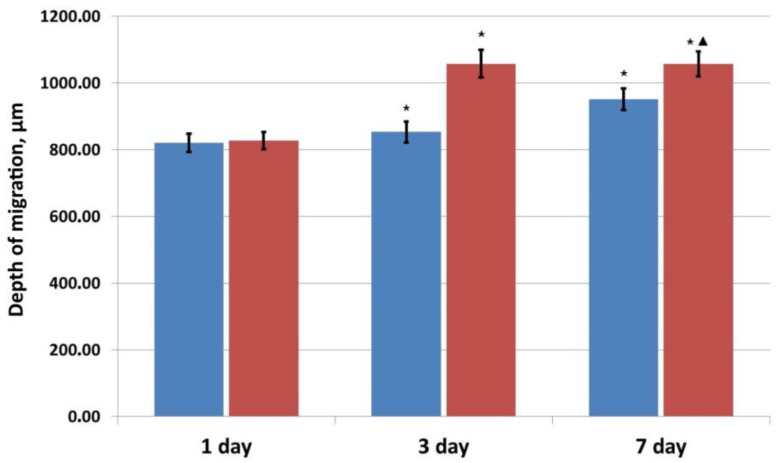
Assessment of the migration of human ASCs into the structure of osteoplastic matrix samples during cultivation. Note: 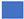
—control series, 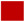
—experimental series; ⋆—*p* < 0.05 compared with Day 1; ▲—*p* < 0.05 comparison, control v experiment (Wilcoxon rank sum test).

Cell migration into the matrix structure was assessed at the same time. It was noted that the cells had migrated into the matrix structure in samples of both series but for only 3 days. Here, migration into the matrix samples containing the factor was slightly greater. During subsequent cultivation up to Day 7, the cells appeared to be fixed at approximately the same depths within samples of both series as they had been on Day 3 of cultivation (Figure 7).

Therefore, considering the results of these three independent experiments, one can note that the matrix with incorporated placental growth factor provided more favorable conditions for the proliferation and migration of human ASCs during the first three days of observation than did the matrix containing no factor. During subsequent cultivation up to Day 7, the number of cells and the depth of migration into the pores of the material in the matrices of both series were almost equal. Based on the data indicating the dynamics of factor release into the medium, it can be assumed that the amount of PLGF releasing into the growth medium was sufficient to influence the ASCs and to enhance their functional activity during the first three days of cultivation. Subsequently, Days 3 to 7, the amount of factor entering the medium decreased significantly, and there was no further stimulatory effect on the activity of the cells within the matrix.

The literature contains descriptions of the influence of placental growth factor on various cell processes: proliferation of progenitor cells, plus the recruitment and activation of monocytes and of hematopoietic stem cells [73,74,75]. In vitro models have proved that secretory PLGF can have an autocrine impact on MSCs at low concentrations, enhancing their osteogenic differentiation, while at higher concentrations, it induces and modulates osteoclastogenesis and angiogenesis [23]. Moreover, it has been demonstrated that PLGF exhibits its maximum pro-osteogenic properties under pathological conditions [48].

Thus, the described properties of placental growth factor make it promising for use in optimizing recovery processes in the case of disease or injury of bone tissue [76]. It is obvious that the optimal use of certain factors in the treatment of bone tissue pathologies is related to their combined use with osteoplastic materials, and such complexes are being actively studied.

Vasculoendothelial growth factor (VEGF) complexes with various other materials have been examined in the greatest detail. For instance, Elaine Quinlan et al. assessed the biological properties of complexes made of alginate particles with VEGF contained within collagen/hydroxyapatite matrices. In an in vitro model, these materials released VEGF for a long time (up to 35 days). Here, the VEGF was released continuously and maintained at a high level during the first 4 days. Then, a decrease in its release was observed, but low factor concentrations could still be detected until the end of the study. Analysis of the effects of such materials on human cells demonstrated their activation of proliferation and differentiation. When implanted in vivo into a rat tibial defect model, the materials enhanced blood vessel formation, which contributed to increased bone regeneration [77].

Another group of researchers [78] studied poly-ε-caprolactone hydroxyapatite/calcium sulfate materials loaded with VEGF. The VEGF could still be detected in the model medium for 14 days and its content there increased during this period. The studied materials encouraged effective adhesion and proliferation of human cells (HMSC, HUVEC). In the rat femoral bone defect model, materials made from poly-ε-caprolactone/hydroxyapatite/calcium sulfate with VEGF were demonstrated to enhance osteoinduction and osteogenesis.

For example, using artificial scaffolds based on alginate, collagen, or hydroxyapatite [79], similar to those used by E. Quinlan and colleagues, Sheehy E.J. et al. (2021) demonstrated that the introduction of PLGF into the scaffolds could be used to deliver the factor and to influence angiogenesis and osteogenesis. The authors registered a dose-dependent effect and a release profile of PLGF with a high rate during the first hours, followed by further moderate release, this result being similar to the data in our study. The scaffolds with PLGF that they used provided reliable bone regeneration in a bone defect model in experimental animals (rats).

The results of these studies suggest that the release profiles of the various factors were most likely influenced not only by the matrix structure and the way it was loaded with any particular growth factor, but also by the nature of the factor itself. However, this hypothesis definitely requires further empirical support.

It is worth noting that, currently, there are many bone-restoring materials of various structures and compositions [80]. Xenomaterials are also included among the currently available, inexpensive, and fairly conveniently used materials. Such materials, primarily xenografts from bovine as well as equine and porcine tissues, are widely used in clinical practice. Of all the xenografts, cancellous bones grafts of bovine origin are recognized as the closest to regenerated human bone [81,82]. Xenografts are advised for use in clinical practice in situations when bone regeneration is required during reconstructive surgical operations [61]. One such material is the osteoplastic matrix, used and described in our study. This material is characterized by the type of highly porous structure required for vascularization and cell colonization. During our assessment of the interactions of human PLGF with human and rabbit ASCs, it was shown that in in vitro experiments, introduction of the factor into the medium resulted in practically no change in the morphological and functional properties of the ASCs. Furthermore, the stimulating effect of placental growth factor on the proliferation of human and rabbit ASCs within 48 h of the start of such interaction was also demonstrated. In this case and in the case of ASC cultivation in a matrix functionalized with human PLGF, activation of proliferation and migration of human ASCs was registered during the early stages of interaction, compared with the control series (matrix with no factor). It can be assumed that the combined action of the factor with the matrix, considering the properties of its composition, porous structure, and surface topography, can optimize the conditions in which the ASCs operate.

To confirm the assumptions made and to evaluate the effectiveness of the modified material, further studies are definitely required, both with in vitro models and in experimental animals.

## 4. Conclusions

This study showed that human placental growth factor at a concentration of 20 ng/mL had no negative impact on the morphological and functional properties of human or rabbit ASCs. Moreover, the human PLGF positively activated proliferation during the first 48 h of exposure in all experiments with both human and rabbit ASCs. In some experiments, the stimulating effect continued for up to 72 h. The possibility of xenogeneic osteoplastic matrix modification by incorporation of PLGF into its structure, and the dynamics of the gradual release of the factor into the model medium were demonstrated. During the cultivation of human ASCs on a functionalized material, its effectiveness in stimulating ASC proliferation and migration was shown. Thus, this work demonstrates both the possibility of changing the biological activity of xenogeneic osteoplastic material by functionalization using human placental growth factor, and confirms the biocompatibility of the modified material in vitro.

This study offers opportunities for further work with the described modification of the material, aimed at optimization of the methods used for incorporation of the factor in order to regulate its optimal dosage and release dynamics.

This experience may be useful in the development of new, biologically active materials and their modifications with placental growth factor.

## Figures and Tables

**Table 1:** Change in the density of human ASCs after interaction with human placental growth factor (cells/cm^2^).

Experiment No.	Interaction Time	24 h	48 h	72 h
Experiment 1 *n* = 6	C	16,368.7 ± 832.7	22,997.7 ± 1104.7 ⋆	21,812.63 ± 1616.0 ⋆
	E	18,072.2 ± 228.3	26,034.43 ± 764.2 ⋆□	26,293.67 ± 250.82 ⋆▲
Experiment 2 *n* = 6	C	7043.7 ± 308.6	10,487.8 ± 340.9 ⋆	18,353.7 ± 903.1 ⋆●
	E	8887.9 ± 252.8 ■	11,354.4 ± 184.4 ⋆□	21,886.7 ± 106.2 ⋆●▲
Experiment 3 *n* = 6	C	13,776.4 ± 593.76	19,998.9 ± 128.29 ⋆	25,701.13 ± 1321.84 ⋆
	E	15,665.03 ± 886.48	22,931.04 ± 633.04 ⋆□	25,664.1 ± 449.01 ⋆●

Note: C—control series, E—experimental series; ⋆—*p* < 0.05 compared with 24 h; ●—*p* < 0.05 compared with 48 h; ■—*p* < 0.05 compared with 24 h, control-experiment; □—*p* < 0.05 compared with 48 h, control-experiment; ▲—*p* < 0.05 compared with 72 h, control-experiment (Wilcoxon rank sum test).

**Table 2:** Change in VEGF-A content in the conditioned medium of human ASCs (in pg/mL) after interaction with PLGF.

Experiment No.	Interaction Time	24 h	48 h	72 h
Experiment 1 *n* = 6	C	400.93 ± 15.18	668.18 ± 36.65 ⋆	1490.97 ± 103.87 **
	E	471.72 ± 25.1	749.14± 42.91 ⋆	1506.23 ± 285.14 **
Experiment 2 *n* = 6	C	296.04 ± 15.58	465.97 ± 28.54 ⋆	592.17 ± 30.51 **
	E	269.39 ± 13.89	497.83 ± 19.16 ⋆	609.21 ± 26.79 **
Experiment 3 *n* = 6	C	208.90 ± 16.61	363.2 ± 37.43 ⋆	539.96 ± 28.22 **
	E	204.98 ± 20.09	377.88 ± 22.85 ⋆	658.71 ± 47.24 **

Note: C—control series, E—experimental series; ⋆—*p* < 0.05 compared with 24 h; **—*p* < 0.05 compared with 48 h, Wilcoxon rank sum test.

**Table 3:** Change in the fibronectin content in the conditioned medium (in ng/mL).

Experiment No.	Interaction Time	24 h	48 h	72 h
Experiment 1 *n* = 6	C	360 ± 20	710 ± 20 ⋆	1240 ± 90 **
	E	400 ± 10	890 ± 70 ⋆	1360 ± 80 **
Experiment 2 *n* = 6	C	1640 ± 20	1900 ± 80 ⋆	1940 ± 20
	E	1720 ± 40	1920 ± 10 ⋆	1900 ± 30
Experiment 3 *n* = 6	C	1430 ± 60	1690 ± 10 ⋆	1830 ± 50 **
	E	1490 ± 40	1730 ± 30 ⋆	1860 ± 30 **

Note: C—control series, E—experimental series; ⋆—*p* < 0.05 compared with 24 h; **—*p* < 0.05 compared with 48 h, Wilcoxon rank sum test.

**Table 4:** Changes in the rabbit ASC densities after interaction with human placental growth factor.

Experiment No.	Interaction Time	24 h	48 h	72 h
Experiment 1r *n* = 6	C	19,398.05 ± 601.02	32,374.54 ± 1798.17 ⋆	73,325.5 ± 2240.14 ⋆●
	E	20,153.54 ± 626.67	45,239.92 ± 2115.74 ⋆□	85,398.87 ± 1182.29 ⋆●▲
Experiment 2r *n* = 6	C	10,369.33 ± 404.33	26,923.23 ± 2430.30 ⋆	68,919.03 ± 2422.16 ⋆●
	E	12,072.87 ± 400.23 ■	44,143.73 ± 1802.12 ⋆□	86,880.2 ± 3493.09 ⋆●▲
Experiment 3r *n* = 6	C	10,125.98 ± 788.97	50,741.3 ± 2746.46 ⋆	50,430.87 ± 2720.10 ⋆
	E	14,442.03 ± 689.93 ■	54,838.35 ± 2548.68 ⋆□	54,861.22 ± 2737.40 ⋆▲

Note: C—control series, E—experimental series; ⋆—*p* < 0.05 compared with 24 h; ●—*p* < 0.05 compared with 48 h; ■—*p* < 0.05 compared with 24 h, control-experiment; □—*p* < 0.05 compared with 48 h, control-experiment; ▲—*p* < 0.05 compared with 72 h, control-experiment (Wilcoxon rank sum test).

**Table 5:** Concentrations of PLGF in the model medium (pg/mL) accumulating over 3 h at different sampling times as it is released from functionalized matrix samples.

Test Points	3 h	24 h	3 Days	7 Days
PGLF in pg/mL (*n* = 9)	2045.30 ± 103.80	176.43 ± 14.93 ⋆	119.96 ± 6.27 ⋆●	96.31 ± 2.68 ⋆●

Note: ⋆—*p* < 0.01 compared with 3 h; ●—*p* < 0.05 compared with 24 h (Wilcoxon rank sum test).

**Table 6:** Evaluation of the cytotoxicity of a PLGF-functionalized matrix.

		Release Period	
		1 Day	7 Days
Control (*n* = 8)	Relative density	0.490 ± 0.020	0.711 ± 0.026
	Relative intensity of cell growth, %	100	100
	Cytotoxicity level	0	0
Extract (*n* = 8)	Relative density	0.424 ± 0.007	0.561 ± 0.009
	Relative intensity of cell growth, %	86.53	78.90
	Cytotoxicity level	1	1
Extract 1:1 (*n* = 8)	Relative density	0.417 ± 0.009	0.521 ± 0.210
	Relative intensity of cell growth, %	85.10	73.28
	Cytotoxicity level	1	2
Extract 1:2 (*n* = 8)	Relative density	0.418 ± 0.005	0.605 ± 0.057
	Relative intensity of cell growth, %	85.30	85.09
	Cytotoxicity level	1	1
Extract 1:4 (*n* = 8)	Relative density	0.459 ± 0.01	0.643 ± 0.048
	Relative intensity of cell growth, %	93.67	90.43
	Cytotoxicity level	1	1
Extract 1:8 (*n* = 8)	Relative density	0.476 ± 0.008	0.622 ± 0.030
	Relative intensity of cell growth, %	97.14	87.48
	Cytotoxicity level	1	1

## Data Availability

Data presented in this study are available on request from the corresponding author.
